# Cystic Fibrosis Lung Disease in the Aging Population

**DOI:** 10.3389/fphar.2021.601438

**Published:** 2021-04-15

**Authors:** Lisa Künzi, Molly Easter, Meghan June Hirsch, Stefanie Krick

**Affiliations:** ^1^ Division of Pulmonary, Allergy and Critical Care Medicine, University of Alabama at Birmingham, Birmingham, AL, United States; ^2^ Epidemiology, Biostatistics and Prevention Institute, Department of Public and Global Health, University of Zürich, Zürich, Switzerland; ^3^ Gregory Fleming Cystic Fibrosis Research Center, University of Alabama at Birmingham, Birmingham, AL, United States; ^4^ Comprehensive Center for Healthy Aging, University of Alabama at Birmingham, Birmingham, AL, United States

**Keywords:** cystic fibrosis, aging, inflammaging, oxidative stress, mitochondrial dysfunction, senescence

## Abstract

The demographics of the population with cystic fibrosis (CF) is continuously changing, with nowadays adults outnumbering children and a median predicted survival of over 40 years. This leads to the challenge of treating an aging CF population, while previous research has largely focused on pediatric and adolescent patients. Chronic inflammation is not only a hallmark of CF lung disease, but also of the aging process. However, very little is known about the effects of an accelerated aging pathology in CF lungs. Several chronic lung disease pathologies show signs of chronic inflammation with accelerated aging, also termed “inflammaging”; the most notable being chronic obstructive pulmonary disease (COPD) and idiopathic pulmonary fibrosis (IPF). In these disease entities, accelerated aging has been implicated in the pathogenesis via interference with tissue repair mechanisms, alterations of the immune system leading to impaired defense against pulmonary infections and induction of a chronic pro-inflammatory state. In addition, CF lungs have been shown to exhibit increased expression of senescence markers. Sustained airway inflammation also leads to the degradation and increased turnover of cystic fibrosis transmembrane regulator (CFTR). This further reduces CFTR function and may prevent the novel CFTR modulator therapies from developing their full efficacy. Therefore, novel therapies targeting aging processes in CF lungs could be promising. This review summarizes the current research on CF in an aging population focusing on accelerated aging in the context of chronic airway inflammation and therapy implications.

## Introduction

30 years ago, mutations in the cystic fibrosis transmembrane regulator (CFTR) gene were characterized as single gene defect leading to the pathology of cystic fibrosis (CF) (Kerem et al., 1989; Tsui, 1995). Since then, our understanding of the pathological processes has largely improved with CFTR targeted modulator therapies in clinical use (Wainwright et al., 2015; Rowe et al., 2017; Taylor-Cousar et al., 2017; Davies et al., 2018; Keating et al., 2018). Nevertheless, progressive lung disease is still the major cause for morbidity and mortality in individuals born with CF (Elborn, 2016). Functional failure of CFTR results in impaired ciliary function, mucus obstruction, bacterial colonization and chronic inflammation (Matsui et al., 1998; Mall et al., 2004; Boucher, 2007). This sets the stage for smoldering chronic infections interrupted by episodic exacerbations due to newly acquired pathogens leading to progressive loss of lung function (Nichols et al., 2008; Elborn, 2016). Elevated levels of pro-inflammatory mediators, reactive oxygen species (ROS), and proteases, which further propagate airway inflammation, can accelerate cellular senescence (Bezzerri et al., 2019).

Major improvements in supportive therapies and the development of CFTR targeted therapies increased life expectancy in CF patients to a median survival of 40 years or more in industrialized nations (Dodge et al., 2007; MacKenzie et al., 2014). Hence, CF is no longer considered primarily a pediatric disease. This entails new challenges, including treatment of CF in an aging population with CF dependent and independent comorbidities. Diabetes, osteopenia and osteoporosis, renal, and vestibulocholear complications from aminoglycoside toxicity, cardiovascular disease, and worsening lung disease are medical issues often emerging in the aging CF population (Hodson et al., 2008; Simmonds et al., 2009). Chronic local and systemic inflammation is a key pathogenic mechanisms suspected of promoting these comorbidities (Nichols et al., 2008; Bezzerri et al., 2019). Aging impairs lung function through several mechanisms, a central being chronic, low-grade inflammation termed “inflammaging” (Franceschi and Campisi, 2014). Accelerated aging has been implicated in several chronic inflammatory lung disease pathologies. As chronic inflammation is a hallmark of CF airway disease, the question arises whether this chronic inflammation may also cause premature aging in CF lung tissue; and if so whether novel therapies targeting “inflammaging” and aging pathomechanisms in CF lungs might prove to be beneficial.

## Hallmarks of Aging in CF Lung Disease

**FIGURE 1:**
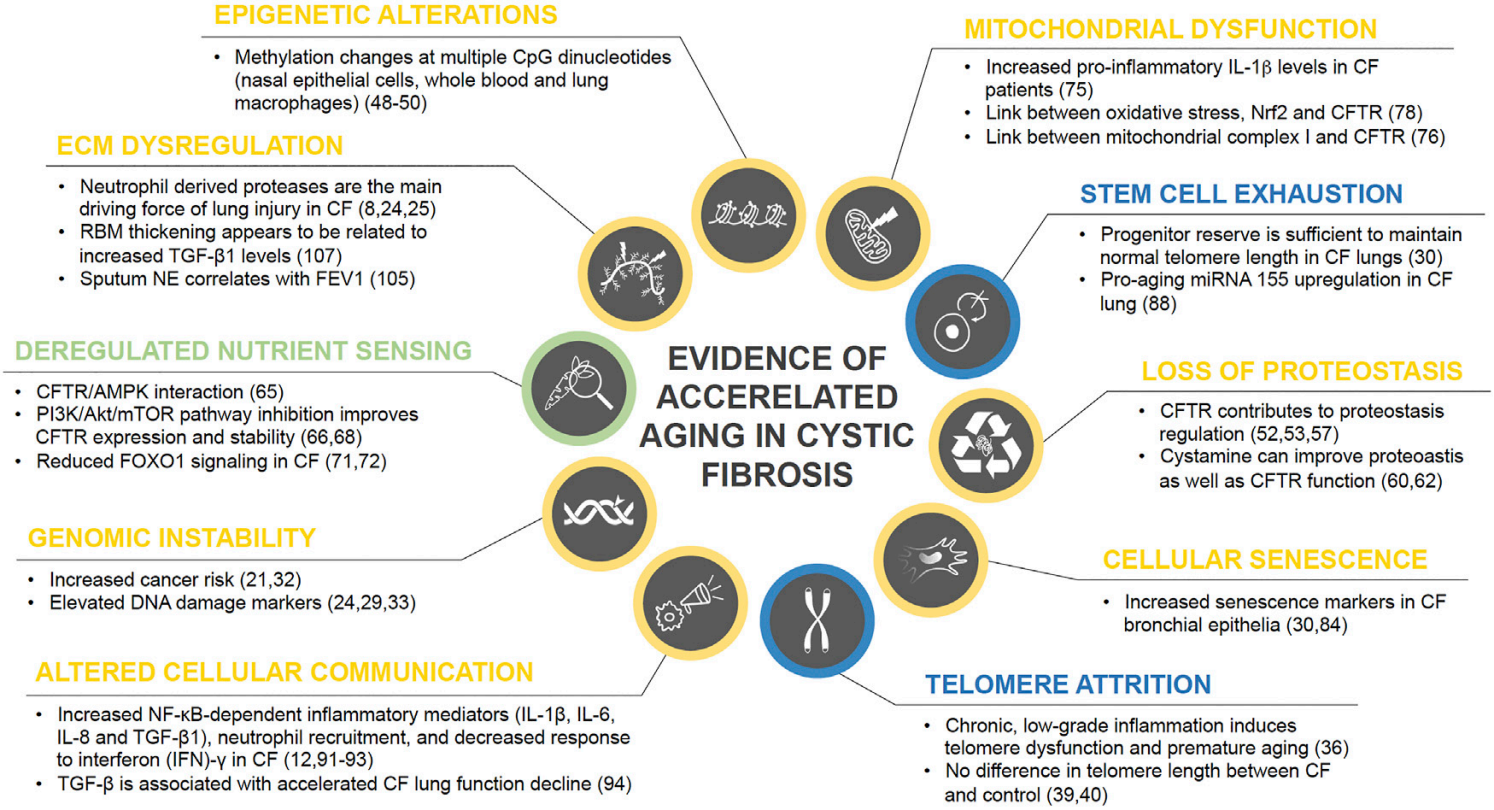
Current state of evidence on accelerated aging processes in CF. Research findings on the role of each of the ten hallmarks of aging in CF disease are summarized and color coded based on their level of evidence with blue = no evidence for involvement in CF pathology, green = weak evidence, yellow = strong evidence.

Physiological aging is associated with considerable changes in lung structure and cell function, giving rise to reduced lung function, pulmonary remodeling, diminished regenerative capacity, and enhanced susceptibility to lung injury, and disease (Cho and Stout-Delgado, 2020). Considering the increased life expectancy of CF patients, the question whether chronic inflammation contributes to accelerated aging becomes more and more relevant. Interestingly, healthy aging lungs show a proinflammatory state with increased neutrophils and IL-8 (Meyer et al., 1998), bearing some similarity with chronic CF lung inflammation. In the following paragraphs we aim to provide an overview on the hallmarks of aging and their potential implications for CF lung disease. Central findings on accelerated aging in CF are also summarized in Figure 1.

### Genomic Instability

Non-transplanted CF patients have a higher risk for lymphoid leukemia, testicular cancer and digestive tract cancers than the general population (Maisonneuve et al., 2013). Similarly, cancer risk in CF lung transplant recipients is more increased than in non-CF recipients, particularly for colorectal cancer, esophageal cancer, and non-Hodgkin lymphoma (Fink et al., 2017). Defects in CFTR themselves have been linked to cancers (Scott et al., 2020). The striking overlap of organ systems with elevated cancer risk and those that are primarily affected by CF-associated mucus hypersecretion, i.e., the respiratory, digestive, and reproductive systems, strongly implicates chronic inflammation as a potential causative factor. In CF airway inflammation, large numbers of activated neutrophils release reactive oxygen species (ROS) and proteases, such as neutrophil elastase, thereby inducing additional ROS production in lung cells (Brown and Kelly, 1994; Aoshiba et al., 2001). In addition, reduced antioxidant defense with systemic glutathione deficiency, reduced vitamin E plasma levels, and increased mitochondrial oxidative stress has been reported in CF patients (Roum et al., 1985; Brown et al., 1995; Starosta et al., 2006; Velsor et al., 2006). Not surprisingly, CF lungs are exposed to significantly higher oxidative stress levels (Starosta et al., 2006). As marker of oxidant-induced DNA damage, urinary 8-hydroxydeoxyguanosine (8-OHdG) was found to be significantly elevated in children with CF (Brown and Kelly, 1994). Further, airway epithelia from CF patients show increased expression of two DNA damage markers (phospho-Histone H2A.X (γH2A.X) and phospho-checkpoint 2 kinase (phospho-Chk2)) (Fischer et al., 2013). However, it would be too simplistic to attribute the increased cancer risk solely to oxidative stress. CFTR has been proposed a tumor suppressor protein and CFTR deficiency is implicated in airway and intestinal cancer progression and poor prognosis (Than et al., 2016; Li et al., 2015; Tu et al., 2016). In summary, aging-associated DNA repair deficiency in combination with increased oxidative damage can lead to accumulating genome damage and thus to genomic instability (Moskalev et al., 2013).

### Telomere Attrition

The proliferative capacity of cells is limited by the gradual loss of their protective telomeric DNA with each division ultimately leading to cellular senescence (Blackburn et al., 2006). Chronic inflammation can not only increase cell turnover, it has also been shown to lead to telomere dysfunction and accelerated aging (Jurk et al., 2014; Gorenjak et al., 2020). Telomeres are highly sensitive to oxidative damage as it is ineffectively repaired causing accelerated telomere loss (Gorenjak et al., 2020; Hewitt et al., 2012). This raises the question whether oxidative stress associated with chronic airway inflammation can cause accelerated telomere shortening in CF patients.

In CF, decreased telomere length in peripheral blood leukocytes has been reported to be associated with disease severity (Lammertyn et al., 2017). However, telomere length was not reduced in explant lungs from CF patients, and no correlation between telomere length and disease severity at the sampling location was found (Everaerts et al., 2018). Similarly, telomere length in CF airway epithelial cells was not significantly different from control donor lungs; only a small subgroup of CF subjects had shorter telomeres (Fischer et al., 2013). Based on the limited scientific data available, we cannot conclude whether telomere attrition is a common feature in CF lungs. Further research is needed to determine its contribution to CF airway disease pathogenesis.

### Epigenetic Alterations

Epigenetic alterations are stable, heritable changes to the chemical DNA structure by alterations in its methylation pattern, post-translational histone modifications, and chromatin remodeling. They regulate gene expression without modifying the DNA sequence (Gonzalo, 1985; Berger et al., 2009). Specific changes in the histone modification pattern such as increased H4K20 and H3K4 trimethylation, and decreased H3K9 methylation as well as H3K27 trimethylation constitute markers of age-associated epigenetic alterations (Gonzalo, 1985; López-Otín et al., 2013). DNA methylation has been shown to play an important role in aging and disease. Global DNA methylation changes over the lifespan and methylation of specific CpG (cytosine guanine) sites correlate with age (Jones et al., 2015; Ashapkin et al., 2017; Levine et al., 2018). Alterations in DNA methylation patterns are associated with cancer, several genetic diseases and inflammatory lung conditions including CF (Robertson, 2005; Scott and De Sario, 2020). In CF, methylation changes were found at multiple CpG dinucleotides in nasal epithelial cells, whole blood and lung macrophages. Interestingly, these epigenetic alterations were preferentially located at genes important for the respective tissue, such as for cell adhesion, immune response or inflammation, and correlated with lung function (Scott and De Sario, 2020; Magalhães et al., 2018; Chen et al., 2018). Though the specific research is yet in its infancy, CFTR has been shown to be epigenetically regulated too which may open up novel therapeutic strategies to restore CFTR dysfunction in CF pathology (Sirinupong and Yang, 2015).

### Loss of Proteostasis

Molecular chaperones and the proteolytic machinery assure continuous proteome renewal protecting cells from accumulation of misfolded or damaged proteins (López-Otín et al., 2013). Loss of adequate proteostasis occurs during the aging process and has been shown to be altered in CF epithelia. There is increasing evidence that CFTR function can regulate the proteostasis network (Villella et al., 2013; Esposito et al., 2016). CFTR carrying the ∆F508 mutation as well as other less frequent mutations does not mature into the fully glycosylated protein. Instead, the misfolded protein is mostly retained at the endoplasmic reticulum, where it is sequestered for degradation (Cheng et al., 1990; Van Goor et al., 2011). ∆F508 overexpression causes ER stress and activates the unfolded protein response *in vitro* leading to decreased wild type CFTR mRNA levels (Bartoszewski et al., 2008). This implicates defective CFTR leads to suppressed CFTR expression. The severity of the clinical disease spectrum varies considerably even within patients carrying the same mutation. Variations in the proteostasis network are suspected to be modifying factors contributing to those differences (Balch et al., 2011). A promising new therapeutic strategy aims at improving protein trafficking by the proteostasis network (Strub and McCray Jr, 2020). The proteostasis regulator cystamine promotes ∆F508-CFTR trafficking to the apical cell membrane and thereby allows it to respond to CFTR potentiators (Luciani et al., 2012). Cystamine combined with epigallocatechin gallate restored CFTR function, reduced lung inflammation and restored autophagy in a phase-2 trial, particularly in patients carrying the ∆F508 mutation (Tosco et al., 2016).

CFTR is not only subjected to degradation by the proteostasis network, it is a key player in its own regulation. Inhibition of CFTR function leads to CFTR protein ubiquitination and drastically reduced stability in the plasma membrane of bronchial epithelial cells (Esposito et al., 2016), with cystamine correcting this deranged proteostasis (Villella et al., 2013). In addition, siRNA depleting CFTR interferes with endosomal trafficking of cell surface proteins demonstrated for transferrin receptor, epidermal growth factor receptor, and CFTR itself (Villella et al., 2013). Hence, insufficient CFTR function derails the proteostasis network in a feed-forward loop fostering its own degradation. Dysfunctional autophagy also appears to contribute to the exaggerated CF lung inflammation. Improving autophagosome clearance attenuates the hyperinflammatory response (Mayer et al., 2013). Oxidative stress from defective CFTR function inhibits ubiquitination and proteasome degradation of the pro-fibrotic tissue trans-glutaminase 2, thereby driving chronic inflammation (Esposito et al., 2016; Luciani et al., 2009). Autophagy restoration by cystamine has been shown to suppress trans-glutaminase 2 and diminish inflammation in CF mice and in patient derived nasal epithelial cells *in vitro*. In summary, loss of proteostasis as an aging hallmark in CF disease has been established by current evidence and might be a valuable target for future therapies.

### Deregulated Nutrient Sensing

Aging is regulated by nutrient-sensing pathways including insulin/insulin-like growth factor (IGF-1), mTOR, AMP kinase, sirtuins, and FOXO transcription factors (Vellai, 2009; Kenyon, 2010). Pro-inflammatory signals are closely integrated in stress and nutrient signaling (Jurk et al., 2014). Hence, both inflammaging and chronic inflammatory conditions such as CF lung disease have the potential to interfere with nutrient sensing signaling pathways. An *in vitro* study identified the α1 (catalytic) subunit of AMP-activated protein kinase (AMPK) as a dominant and novel protein interacting with CFTR demonstrating a potential link between transepithelial transport and cell metabolic state (Hallows et al., 2000).

Oxidative stress as seen in CF airway inflammation leads to increased pro-growth signaling by the mTOR pathway (Chen et al., 2011). So far, only little is known about deregulated nutrient sensing in CF lung disease. CFTR has been found to interact with mTOR signaling pathway components, which can be affected by CFTR mutations such that ∆F508 CFTR exhibits a specific interactome different from wild type CFTR (Pankow et al., 2015; Reilly et al., 2017). mTOR activity in CF bronchial epithelial cells is upregulated, and PI3K/Akt/mTOR pathway inhibition improves CFTR expression and stability (Reilly et al., 2017). FOXO transcription factors down stream of insulin/IGF-1 signaling (IIS) regulate cellular processes involved in stress resistance, metabolism as well as cell cycle arrest, and are central to IIS attenuation-mediated lifespan expansion (Martins et al., 2016). FOXO1 and 3 have been shown to regulate innate immune mechanisms in respiratory epithelia in response to bacterial infections (Seiler et al., 2013). In a human CF bronchial epithelial cell line, reduced FOXO1 was found related to loss of CFTR function (Smerieri et al., 2014). Interestingly, four miRNAs that are predicted FOXO1 regulators were differently expressed in CF patient sera (Montanini et al., 2016). However, the implications of altered FOXO transcription factor signaling in terms of accelerated aging in CF patients remains unclear and calls for further investigation.

### Mitochondrial Dysfunction

Age-associated mitochondrial dysfunction is most commonly caused by increased ROS ultimately leading to cell senescence (Chapman et al., 2019). Excessive oxidant levels from dysfunctional mitochondria can trigger inflammatory cytokine release inducing chronic inflammation and progression of airway diseases such as CF (Prakash et al., 2017). In addition, as cells age, DNA damage occurs and activates DNA damage response pathways. These chronically stimulated pathways cause mitochondrial stress leading to increased release of mitochondrial damage-associated molecular patterns (DAMPs). DAMPs along with ROS production, and/or ATP and K^+^ efflux contribute to NLRP3 inflammasome activation (Prakash et al., 2017; dos Santos et al., 2012). This leads to caspase-1 activation and release of the proinflammatory cytokines IL-1β and IL-18, initiating a cycle of chronic inflammation, disease progression, and further accumulative damage to mitochondria resulting in accelerated aging (dos Santos et al., 2012). Recent research is showing that mitochondrial dysfunction could be involved in disease progression in CF. Cells with impaired CFTR function exhibit reduced mitochondrial complex I activity (Valdivieso et al., 2012). And increased IL-1β levels are very common among CF patients, which has been linked to inflammation triggered by underlying chronic *Pseudomonas aeruginosa* infections (Rimessi et al., 2015). As discussed, elevated IL-1β is not only a sign of inflammation, but also a hallmark of mitochondrial dysfunction. These two intrinsically connected hallmarks of aging both contribute to disease progression in CF. Furthermore, it will be of benefit to assess whether the triple CFTR modulator therapy affects these pathogenetic mechanisms favorably. A recent study has shown that CFTR modulation can restore Nrf2 phosphorylation, a major regulator of oxidative balance and inflammatory signaling (Borcherding et al., 2019).

### Cellular Senescence

Accumulation of senescent cells is seen in normal aging and chronic pulmonary disease (Jeyapalan et al., 2007; Meiners et al., 2015). Senescence is triggered by a range of insults such as oxidative stress, DNA damage, telomere shortening, and inflammation, and is associated with a characteristic secretory profile termed senescence-associated secretory phenotype (SASP) (Naylor et al., 2013; Jurk et al., 2014). This includes release of proinflammatory mediators, growth factors, and matrix-remodeling proteases that may perpetuate inflammatory processes resulting in further accumulation of senescent cells (Naylor et al., 2013; Parikh et al., 2019). In CF, the liquid lung lining layer contains high amounts of neutrophil elastase that has been shown to trigger cell cycle arrest through elevated p27^Kip1^ expression resulting in G1 arrest in normal human bronchial epithelial cells *in vitro* (Nakamura et al., 1992; Fischer et al., 2007). Cell cycle arrest may lead to cellular senescence (Fischer et al., 2013; Naylor et al., 2013). Indeed, a study by Fischer and colleagues confirmed airway epithelia from CF lungs have increased expression of the senescence markers p16^INK4a^, γH2A.X, and phospho-Chk2 (Fischer et al., 2013). Their study showed that neutrophil elastase increases p16 expression resulting in inhibition of cyclin-dependent kinase 4 activity *in vitro* (Fischer et al., 2007). This suggests cellular senescence due to excessive neutrophil elastase release in CF lungs may contribute to accelerated aging. A recent review concluded there was consistent data supporting that cellular senescence may also be involved in CF lung disease, but the exact mechanisms leading from loss of CFTR function to cellular senescence and the precise role in CF lung disease remain unknown (Bezzerri et al., 2019).

### Stem Cell Exhaustion

Lung tissue has a low steady-state cell turnover with the ability to increase proliferation of stem/progenitor cells in response to injury (Hogan et al., 2014). Aging is associated with a progressive decline of stem/progenitor cells that maintain homeostatic and regenerative capacity (Oh et al., 2014). Chronic inflammation is considered a main factor accelerating the deterioration of stem cell function with transforming growth factor (TGF)-β and ROS accumulation as important pathological factors (Oh et al., 2014). miRNA-155 is upregulated in aging and has been suggested to contribute to inflammation-associated stem cell dysfunction (Teramura and Onodera, 2018). High expression of this specific miRNA has been found in CF lung epithelial cells and circulating neutrophils (Bhattacharyya et al., 2011). Further, chronic airway inflammation in combination with recurrent exacerbations causes recurrent tissue damage thereby increasing the need for stem cell proliferation. This may exceed the supply the stem cell niche is capable of providing resulting in its depletion in CF lungs (Mora and Rojas, 2013). To this point, there are no reports on the exact role of stem cell exhaustion in CF lung disease. One study observed no telomere shortening in CF airway epithelial cells leading to the conclusion that the epithelial stem/progenitor reserve is sufficient to maintain normal telomere length despite enhanced cell turnover (Fischer et al., 2013). But this does not exclude an involvement of stem cell exhaustion in accelerated aging in CF lungs, and further studies are needed to draw definite conclusions.

### Altered Cellular Communication

In addition to chronic lung inflammation, CFTR deficiency has been reported to causes abnormalities in diverse signaling pathways. Functional CFTR has been reported to downregulate NF-κB activity and CF associated hyper-inflammation may represent a consequence of insufficient inhibition of NF-κB signaling (Hunter et al., 2010). Chronic, progressive low-grade inflammation promoted by NF-κB can cause premature aging (Jurk et al., 2014). CF airway inflammation is associated with excessive production of NF-κB- dependent inflammatory mediators such as interleukin-1β (IL-1β), IL-6, IL-8, and TGF-β1, neutrophil recruitment, and decreased response to interferon (IFN)-γ as well as further abnormalities in various signaling pathways (Nichols et al., 2008; Harris et al., 2009; Peterson-Carmichael et al., 2009; Lara-Reyna et al., 2020). The profibrotic cytokine TGF-β is considered a pro-aging factor and is associated with accelerated lung function decline in CF patients (Arkwright et al., 2000). Several reports show that TGF-β causes CFTR dysfunction (Manzanares et al., 2015; Snodgrass et al., 2013; Sun et al., 2014). Chronic bacterial infections stimulate persistent IL-8 release from airway epithelia (McCuaig and Martin, 2013). Attracted neutrophils further potentiate airway inflammation by releasing high concentrations of inflammatory mediators such as TNFα and IL-8, oxidants, and proteases (Petit-Bertron et al., 2008). Neutrophil elastase promotes CFTR degradation and dysfunction by calpains (Le Gars et al., 2013). Recent drug developments have focused on restoring CFTR function; these therapies significantly improve lung function and reduce exacerbations in CF patients (Wainwright et al., 2015; Rowe et al., 2017; Davies et al., 2018; Keating et al., 2018). However, sustained airway inflammation leads to degradation and increased turnover of CFTR, hindering the targeted therapies from developing their full potential by inducing a state of accelerated aging at the tissue level (Rowe et al., 2014). Oxidative stress from ROS associated with neutrophilic airway inflammation can lead to accumulation of modified or damaged biomolecules impairing their function. Oxidatively damaged cellular structures can act as DAMPs that are recognized by receptors of the innate immune system, i.e., Toll-like-receptors (TLRs) and the NLRP3 inflammasome (Meiners et al., 2015). Hence, oxidative stress can further enhance cytokine production in the ongoing inflammation and maintain the proinflammatory state in inflammaging (Franceschi and Campisi, 2014; Meiners et al., 2015).

### Extracellular Matrix Dysregulation

ECM remodeling in lung diseases is not only a consequence of tissue injury, it also contributes to disease progression by impaired repair mechanisms (Parker et al., 2014). The neutrophilic inflammation in CF is accompanied by protease/antiprotease imbalance with increased neutrophil elastase and matrix metalloprotease-9 and reduced tissue inhibitor of metalloprotease-1 (Gaggar et al., 2007). While proteolytic enzymes are beneficial for tissue repair, excessive release due to chronic inflammation may overwhelm anti-protease activity causing airway remodeling and obstruction (Gaggar et al., 2011). Neutrophil derived proteases are the main driving force of lung injury in CF (Elborn, 2016) and sputum neutrophil elastase correlates with FEV_1_ in children with CF (Sagel et al., 2002). Furthermore, neutrophil elastase and proteolytic collagen and elastin breakdown products play a pathogenic role in fibrotic lung remodeling (Chua and Laurent, 2006). Ultrastructural evidence for ECM degradation includes lysis of elastic and collagen fibers, loss of arborescent elastic network distribution and alterations in the reticular basement membrane structure (RBM) (Durieu et al., 1998; Hilliard et al., 2007; Regamey et al., 2011). In CF, RBM thickening appears to be related to increased TGF-β1 levels (Hilliard et al., 2007). The elastic network reduction combined with increased collagen deposition in bronchial walls of CF patients resembles fibrotic ECM changes in aging lungs (Hilliard et al., 2007). Together, these data suggest that ECM dysregulation plays an important role in accelerated lung aging in CF.

## Therapeutic Implications

### Current Effective CF Therapies Targeting Chronic Inflammation

Various drugs targeting inflammation have been studied in CF since 1990, but only few are recommended for clinical use. Due to the length of this review, only significant ones will be discussed here.

Corticosteroids were one of the first anti-inflammatory drugs studied as a chronic therapy attenuating CF airway inflammation (Auerbach et al., 1985; Eigen et al., 1995). Although beneficial effects on lung function were shown, overall adverse effects outweighed the benefits and long-term use of systemic corticosteroids to slow lung function decline is currently not recommended (Flume et al., 2007). Several studies investigated the effects of inhaled corticosteroids (ICS) on lung function decline, but their anti-inflammatory effect was never confirmed (Ren et al., 2008; De Boeck et al., 2011). The CF Foundation therefore is advising against the use of long-term ICS in patients with CF older than 6 years without coexistent asthma or allergic bronchopulmonary aspergillosis (Flume et al., 2007).

Nonsteroidal anti-inflammatory drugs (NSAIDs) have been studied as therapeutic options for CF due to similar properties as corticosteroids, but fewer adverse effects. Ibuprofen has specific activity against neutrophils and twice-daily high-dose ibuprofen use was linked to a slower lung function decline, a decreased exacerbation frequency and less weight loss in the pediatric CF population (Konstan et al., 1995; Konstan et al., 2003; Konstan et al., 2007; Lands et al., 2007). Nevertheless, ibuprofen is not widely used, but the CF Foundation expert panel recommends its use for CF patients with mild lung disease (Flume et al., 2007).

**FIGURE 2:**
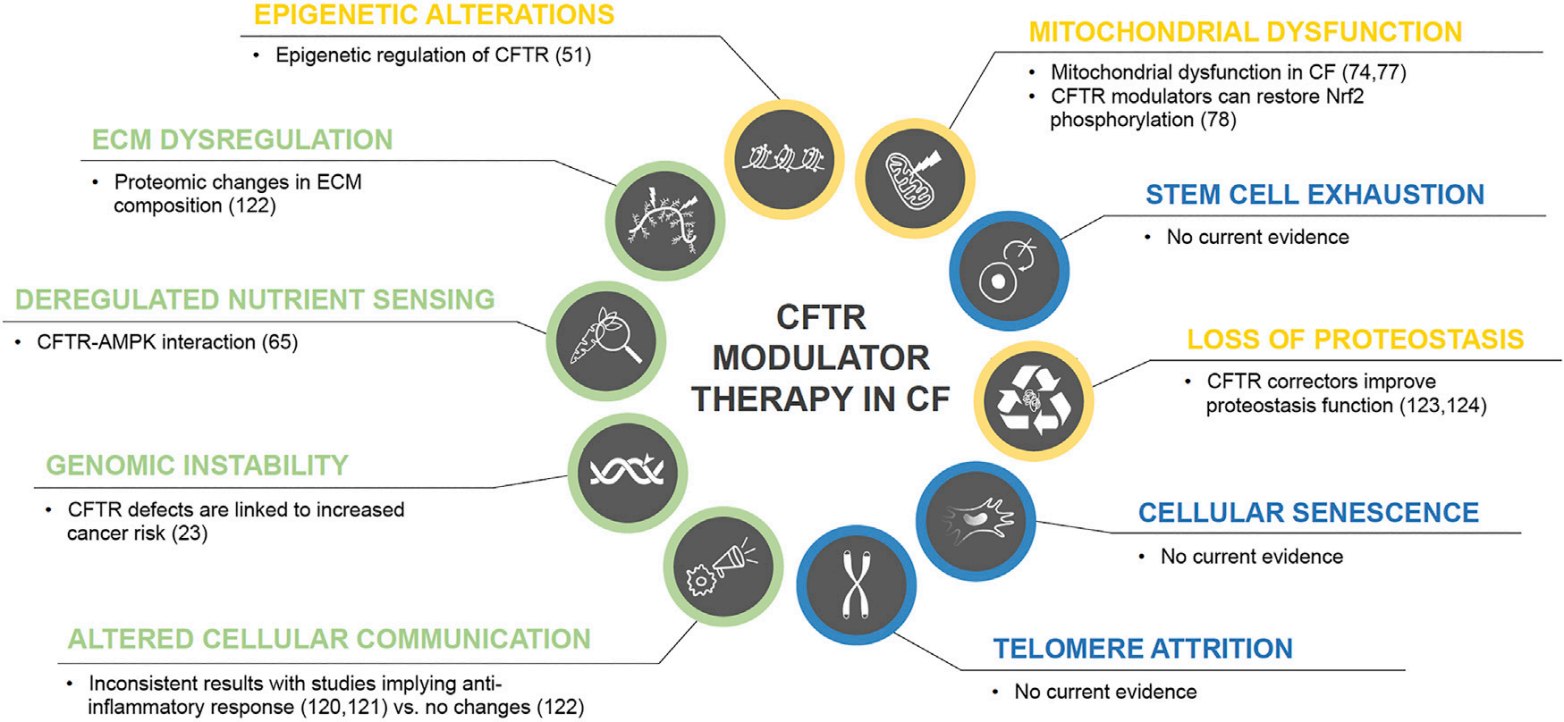
CFTR modulator therapy in CF and potential effects on the hallmarks of aging. Color code for current level of evidence with blue = no evidence for involvement in CF pathology, green = weak evidence, yellow = strong evidence.

CFTR modulator therapy has demonstrated its efficacy and beneficial effect on restoration of CFTR function for approximately 90% CF genotypes (Wainwright et al., 2015; Rowe et al., 2017; Davies et al., 2018; Keating et al., 2018; Middleton et al., 2019). Interestingly, CFTR dysfunction has been linked to contribute to chronic inflammation as well as other hallmarks of aging, and two recent studies have demonstrated an anti-inflammatory effect of CFTR modulators (Jarosz-Griffiths et al., 2020; Hisert et al., 2017). A recent observational study though did not show any significant alterations in sputum inflammatory markers, but changes in ECM using whole proteome analysis (Kopp et al., 2020). Therefore, the validation of potential anti-inflammatory effects of CFTR correctors needs further investigation. CFTR correctors also have been shown to act as proteostasis regulators (Lopes-Pacheco et al., 2015; Lopes-Pacheco et al., 2016) and there is evidence that CFTR modulators can regulate mitochondrial dysfunction. Figure 2 summarizes current evidence of CFTR modulators and their link to aging hallmarks.

### Potential Novel CF Therapies

Modulation of ion channels other than CFTR has been evaluated as potential therapies. Most recently, potentiation of TMEM16A using ETX001 significantly increased the Ca^2+^-activated Cl^−^ channel activity and anion secretion both *in vitro* and *in vivo* ovine models leading to improved mucociliary clearance without impacting calcium signaling (Danahay et al., 2020). Whether ETX001 also affects airway inflammation will be of great interest.

Anti-inflammatory cytokines and antibodies targeting cytokines may be valuable novel CF therapies. IL-10 has been demonstrated to terminate the inflammatory response and is deficient in CF patients (Bonfield et al., 1999). And its administration is anti-inflammatory in Pseudomonas infected mice (Chmiel et al., 1999; Soltys et al., 2002). Treatment with the anti-inflammatory cytokine IFN-γ was tested in a multicenter clinical trial but did not show any beneficial effect on pulmonary function or inflammatory sputum markers (Moss et al., 2005). This is in line with the above-mentioned reduced response of CF airways to IFN-γ. As an alternative to the treatment with anti-inflammatory cytokines, various blocking antibodies targeting pro-inflammatory cytokines have been assessed. Anti-IL-17 antibodies have been shown to decrease neutrophil recruitment in murine airways when exposed to lipopolysaccharide (Ferretti et al., 2003). Anti-IL-17 has been tested clinically in rheumatoid arthritis and psoriasis (Ly et al., 2019; Taams, 2020) and might be a potential novel therapeutic strategy for CF-associated airway inflammation. Anti-ICAM-1 and -IL-8 have been assessed preclinically, but never reached the clinical stage. Because of high concentrations of LTB_4_ in CF airways (Konstan et al., 1993) BIIL 284 BS (amelubant), a specific LTB_4_ receptor antagonist, has been tested in CF. Due to serious pulmonary adverse events, the clinical trial was terminated early (Konstan MW et al., 2005).

Fatty acid modulation may proof beneficial as alterations in fatty acid concentrations contributing to airway inflammation have been demonstrated in CF such as increases in arachidonic acid and decreases in docosahexaenoic acid (DHA) (Freedman et al., 1999; Horati et al., 2020). Cumulative data from *in vitro* studies and some small, non-placebo controlled trials using oral DHA supplementation imply this may be beneficial for pulmonary function preservation (Beharry et al., 2007; Van Biervliet et al., 2008; Aldámiz-Echevarría et al., 2009). To validate these results, larger placebo-controlled trials will be needed. Furthermore, Myriocin, an inhibitor of sphingolipid synthesis, induced changes in a transcriptional program of cell metabolism *in vitro* in CF airway epithelial cells. Therefore, the authors speculated that sphingolipid *de novo* synthesis could attenuate chronic inflammation, optimize energy supply, and anti-oxidant responses, implying a novel future therapeutic strategy for CF (Mingione et al., 2020)

Inhibiton of NF-κB activation has been demonstrated by both ibuprofen at high doses and IL-10. Furthermore, NF-κB signaling can be attenuated by glitazones through peroxisome proliferator activating receptor (PPAR) elevation (Zingarelli et al., 2003; Ruan et al., 2003) and there seems to be a deficiency of PPAR in CF (Perez et al., 2008). Both tiglitazone and troglitazone can activate PPAR in the CF epithelium and attenuate the inflammatory response to *P. aeruginosa* (Perez et al., 2008). One clinical trial using glitazones has not shown a beneficial effect towards inflammation, but this trial was very small and did not assess lung function outcomes (Konstan MW et al., 2009). Long term use of Azithromycin has not only been shown to improve functional outcomes, but some of the underlying mechanisms can be attributed to STAT and NF-κB inhibition (Haydar et al., 2019; Nichols et al., 2020). In summary, targeting NF-κB signaling in CF disease seems to be an attractive future therapeutic direction.

Inhibition of the NLRP3 Inflammasome has also been discussed as a potential anti-inflammatory strategy (Scambler et al., 2019). A recent report utilized MCC950 as a specific NLRP3 inhibitor *in vivo* in CF animal models, which resulted in significantly reduced airway inflammation and improved *Pseudomonas* clearance (McElvaney et al., 2019).

Statins exhibit anti-inflammatory effects through various mechanisms including inhibition of neutrophil migration, RhoGTPase/increase in nitric oxide (NO)/IL-8 production and other proinflammatory cytokines as well as increase of PPAR transcription (Dunzendorfer et al., 1997; Kraynack et al., 2002; Zelvyte et al., 2002). Agents that can increase NO, such as arginine, have been studied, since it has been shown that there is increased arginase activity in blood and sputum of CF patients. This leads to degradation of L-arginine, which is a substrate for NO production (Grasemann et al., 2005a). A small pilot study assessed oral supplementation with L-arginine in CF patients, which led to increased exhaled NO (Grasemann et al., 2005b). In order to show efficacy, large prospective clinical trials are needed.

The antioxidant N-Acetyl cysteine (NAC) is frequently used as a mucolytic, but has spiked recent interest as antioxidant due to its ability to increase glutathione levels and inhibit H_2_O_2_-induced damage. Supplementation of oral NAC was linked to significantly increased blood glutathione levels and decreased neutrophils, IL-8, and elastase activity in sputum of CF patients (Tirouvanziam et al., 2006) A randomized double-blind placebo-controlled trial including 70 CF patients for 24 weeks showed stability or a slight increase in spirometric lung function in NAC treated participants compared to the control group (Conrad et al., 2015). A second open-label randomized controlled trial demonstrated a non-significant improvement in lung function in adults with CF and chronic Pseudomonas infection (Skov et al., 2015). P3001, a mucolytic agent was also assessed and directly compared with NAC in a more recent study using both *in vitro* and *in vivo* CF models. Results indicated that P3001 acted faster and was more effective than NAC and DNAse, but investigators did not study its anti-oxidant or anti-inflammatory properties (Ehre et al., 2019). Since glutathione is transported by CFTR (Linsdell and Hanrahan, 1998), CFTR defects could potentially decrease levels of this antioxidant in the airway epithelium increasing susceptibility to oxidative stress. Indeed, decreased glutathione levels have been observed in CF mouse models and patients (Roum et al., 1985; Velsor et al., 2001). A small pilot study using twice daily treatment of aerosolized glutathione in CF patients led to a reduction in superoxide production, but no changes in oxidative stress markers in bronchoalveolar lavage fluid (Bishop et al., 2005; Hartl et al., 2005). A more recent Cochrane meta-analysis summarized antioxidant therapy approaches in CF and concluded that there does not seem to be a beneficial effect of antioxidant micronutrients on clinical outcomes; but oral supplementation with glutathione showed some benefit to lung function, nutritional status, and decrease in oxidative stress (Ciofu et al., 2019). Nevertheless, due to concurrent treatments including intensive antibiotic therapies, there is not sufficient evidence yet justifying their use in the CF population without larger trials and longer follow up.

Therapeutic use of protease inhibitors has been investigated in CF since 1990. Several groups demonstrated that α_1_-antitrypsin delivered as an aerosol formulary decreases inflammatory markers and neutrophils (McElvaney et al., 1991; Griese et al., 2007). rSLPI and EPI-hNE4 are two neutrophil elastase inhibitors, which have shown beneficial effects in CF, but large cohort trials are still lacking (McElvaney et al., 1993; Grimbert et al., 2003).

Other potential anti-inflammatories are also being investigated. SB-656933, a CXCR2 antagonist, has shown a trend toward attenuating airway inflammation (Moss et al., 2013). Low-dose cyclosporin A was characterized as a potential steroid sparing agent in a small case series (Bhal et al., 2001). Furthermore, methotrexate was studied as a potential anti-inflammatory agent in cystic fibrosis, but studies were inconclusive with one showing beneficial effects on lung function and total serum immunoglobulin levels, whereas another study showed less tolerance and increased exacerbations (Ballmann et al., 2003; Oermann and Wheeler C, 2007).

Micro RNAs as a potential anti-inflammatory approach have recently become a focus of interest. mi-RNAs have been implicated in various diseases including cystic fibrosis (Bardin et al., 2019; Bartoszewska et al., 2017). Vencken et al. suggested the use of nebulized lipid-polymer hybrid nanoparticles for the delivery of a therapeutic anti-inflammatory microRNA, miR-17, to reduce IL-8 secretion, which was successfully tested *in vitro* (Vencken et al., 2019) In addition, targeting miRNAs to restore CFTR activity has also been suggested as a novel therapeutic strategy (De Santi et al., 2020). Please see Table 1 for a summary of the evidence for accelerated aging in CF shown in in vitro and in vivo studies.

**TABLE 1:** Summary of *in vitro*, animal and human studies pointing towards an involvement of accelerated aging in CF disease.

*In vitro* studies			
Hallmark of aging	Cell culture model	Main findings	Ref
Genomic instability	Human fetal lung fibroblast cell line IMR-90, primary human bronchial epithelial cells, and lymphocytes isolated from human blood	ROS and proteases from activated neutrophils increase ROS in mitochondria and cytoplasm, which is associated with oxidative cell injury and death	(Aoshiba et al., 2001)
	Human CF lung epithelial cell line IB3-1 (compound heterozygot for ∆F508 and 128W2X) and isogenic stably wild-type (wt) CFTR transfected C38 cells	Decreased mitochondrial reduced glutathione (GSH) and increased ROS in CFTR deficient human lung epithelial cells	(Velsor et al., 2006)
	CFTR overexpression and knockdown in A549 cell line	Inhibition of CFTR activity promotes epithelial-mesenchymal transition through the uPA/uPAR pathway	(Li et al., 2015)
Loss of proteostasis	COS-7 cell line	Mutated CFTR such as ∆F508 remains in the endoplasmatic reticulum (ER) and is sequester for degradation	(Cheng et al., 1990)
	Calu-3 (wt and stably ∆F508-CFTR transfected) and CFPAC-1 (endogenous ∆F508-CFTR) cell line	∆F508-CFTR overexpression causes ER stress and activates the unfolded protein response leading to decreased wt CFTR mRNA and protein maturation	(Bartoszewski et al., 2008)
	Normal and CFTR mutated CFBE41° bronchial epithelial cells, primary human bronchial/tracheal epithelial cells and HeLa cells	Functional CFTR controls its own surface expression in a positive feed-forward loop through its effects on the proteostasis network. siRNA depleting CFTR interferes with endosomal trafficking of cell surface proteins. Proteostasis regulator cystamine corrects the deranged proteostasis	(Villella et al., 2013)
	FRT cell line, HEK-293 cells, and primary human bronchial epithelial (HBE) cells	CFTR corrector VX-809 improves F508del-CFTR processing in the ER, leading to plasma residence time and susceptibility to proteolysis similar to normal CFTR.	(Van Goor et al., 2011)
	Human normal and CF bronchial epithelial cell lines (CFBE41o-, IB3-1, 16HBE14o-), ex-vivo cultures of nasal polyp mucosal biopsies and brushed nasal epithelial cells from ∆F508 homozygous patients and matched controls	Proteostasis regulators cystamine and EUK-134 (superoxide dismutase/catalase-mimetic) improve ∆F508-CFTR trafficking and stability at the epithelial cell surface by overexpressing BECN1 and depleting SQSTM1. This facilitates its response to CFTR potentiators and suppresses inflammation	(Luciani et al., 2012)
	IB3-1 and isogenic stably rescued C38 cells and peripheral blood mononuclear cells (PBMCs) from pediatric CF patients and healthy controls	Dysfunctional autophagy appears to contribute to the exaggerated CF lung inflammation. Improving autophagosome clearance attenuates the hyperinflammatory response	(Mayer et al., 2013)
	IB3-1 and isogenic stably rescued C38, 16HBE and A549 cell line, *ex vivo* cultures of nasal polyp mucosal biopsies from CF patients and controls	Defective CFTR function generates oxidative stress that leads to PIASy mediated tissue trans-glutaminase 2 (TG2) SUMOylation inhibiting its ubiquitination and proteasome degradation. TG2 inhibition increases NF-κB inhibitor Ikκα	(Luciani et al., 2009)
Deregulated nutrient sensing	CHO (wt and stably expressing CFTR), T84 and Calu-3 cell line, and Xenopus oocytes	AMPK and CFTR are endogenously expressed in the same tissue types and have been found to interact with each other leading to CFTR phosphorylation and altered CFTR Cl^−^ conductance. This may represent a link between transepithelial transport and cell metabolic state	(Hallows et al., 2000)
	CF human bronchial epithelial cell line CFBE41o- (∆F508 mutation) and isogenic HBE41o- cells (wt CFTR)	∆F508-CFTR interactome differs highly from its wt counterpart including differences in the mTOR, JAK/STAT and several other pathways, showing the catastrophic effects from one misfolded protein on protein-protein interactions	(Pankow et al., 2015)
	CFBE41o- and HBE41o- cells	Inhibition of the PI3K/Akt/mTOR pathway leads to improved CFTR stability, while select inhibitors of this pathway leads to restored autophagy and reduced ∆F508-CFTR aggregates	Reilly et al. (2017)
	CFBE41o- and 16HBE14o- cells	Reduced level of transcription factor FOXO1 and β2 arrestin, along with increased ERK1/2 in CF cells. FOXO1 reduction is linked to loss of CFTR function and increased after insulin-like growth factor 1 (IGF-1) administration. Reduced FOXO1 may explain insulin insensitivity in CF, with IGF-1 constituting a potential treatment of CF-related diabetes	(Smerieri et al., 2014)
	CFBE41o-, 16HBE14o-, and IB3-1 cells	Altered transcriptional profile of miRNAs in CF cells, four of which are potential FOXO1 regulators. These four miRNAs are also differentially expressed in CF patients, and dependent on genotype and glucose tolerance state. This may explain some of the variability in metabolism among CF patients	(Montanini et al., 2016)
Mitochondrial dysfunction	IB3-1 cell line	*Pseudomonas eruginosa* induced inflammation shows importance of mitochondria in the pro-inflammatory condition in CF including their role in Ca^2+^ signaling along with NLRP3 recruitment and activation	Rimessi et al. (2015)
	CF and non-CF HBE cells	∆F508-CFTR correctors recover diminished function of the major redox balance and inflammatory signaling regulator Nrf2, inducing its nuclear translocation and transcription of target genes. Nrf2 rescue is dependent on CFTR function	(Borcherding et al., 2019)
Cellular senescence	Normal HBE cells	Neutrophil elastase (NE) triggers cell cycle arrest through elevated p27Kip1 expression resulting in G1 arrest in normal HBE cells	(Fischer et al., 2007)
	Normal HBE cells	NE increases p16 expression and decreases CDK4 activity in HBE cells, which may explain how NE treatment triggers cell cycle arrest	(Fischer et al., 2013)
Stem cell exhaustion	HBE cells	No general telomere shortening in CF HBE cells leading to the conclusion that progenitor reserve is sufficient to maintain normal telomere length despite enhanced cell turnover	(Fischer et al., 2013)
	IB3-1 and control CFTR repaired IB3-S9 cells	CF lung epithelial cells hyperexpress miRNA-155, also upregulated in aging. This activates PI3K/Akt signaling through reduced SHIP1. Resulting activation of downstream MAPKs stabilizes IL-8 mRNA and thus increases IL-8 expression promoting inflammation	(Bhattacharyya et al., 2011)
Altered cellular communication	NCI-H441 and 16HBE14o- cells	Functional CFTR downregulates NF-κB activity. CF associated hyper-inflammation may represent a consequence of insufficient inhibition of NF-κB signaling	Hunter et al. (2010)
	HBE cells	TGF-β_1_ decreases expression of the γ-subunit LRRC26 of the apically located large-conductance Ca^2+^- and voltage-dependent K^+^ (BK) channels. Thereby, TGF-β_1_ reduces BK activity, airway surface liquid volume and ciliary beat frequency	Manzanares et al. (2015)
	Normal and homozygous △508-CFTR HBE cells	TGF-β_1_, which is frequently elevated in CF patients, reduces CFTR mRNA and protein level in non-CF HBE cells. TGF-β_1_ also impairs functional rescue of △508-CFTR suggesting it may interfere with therapies aiming at correcting the processing defect of △508-CFTR.	Snodgrass et al. (2013)
	T84 cell line and HBE cells	TGF-β reduces calcium activated chloride conductance (CaCC) and CFTR-dependent chloride currents. It reduces expression and activity of TMEM16A and CFTR, and reverses △508-CFTR correction by VX-809. Inhibition of Smad3 and p38 MAPK partially reverses TMEM16A and CFTR downregulation	Sun et al. (2014)
	NCI-H292 cell line infected with wt or △508-CFTR	NE promotes degradation of wt and △508-CFTR through activation of intracellular calpain protease causing loss of channel function	Le Gars et al. (2013)

*In vivo* animal studies			
Hallmark of aging	Animal model	Main findings	Ref
Genomic instability	Congenic CFTR KO strains (S489X and FABP) and C57B6 control mice	Decreased mitochondrial GSH in lungs of CFTR-knockout mice, and increased mitochondrial DNA oxidation and oxidative stress	Velsor et al. (2006)
	Athymic balb/c mice injected with CFTR knockdown or control A-549 cells	CFTR status affects cell invasion and migration. No difference in primary tumor growth between control and CFTR-knockdown A549-injected mice, but increased lung metastasis and increased tumor burden by CFTR-knockdown cells	Li et al. (2015)
Telomere attrition	Nfkb1^-/-^ mice (C57B1/6 background) and fibroblast cultures from ear clippings, p55^△ns/△ns^ mice, and late-generation (F3-F4) terc^−/-^ mice bred from B6/Cg-TERC^tm1Rdp^/J	Chronic, low-grade inflammation induces telomere dysfunction, senescence, impaired tissue regeneration and premature aging. Conversely, telomere dysfunction leads to a pro-inflammatory state. Anti-inflammatory or antioxidant treatment blocks accumulation of telomere-dysfunctional senescent cells in nfkb1^-/-^ tissues	Jurk et al. (2014)
Loss of proteostasis	Cftr^F508del/F508del^ mice (129/FVB outbred background) and wt littermates	Autophagy restoration by cystamine treatment, BECN1 overexpression and SQSTM1 depletion all considerably increase ∆F508-CFTR at the epithelial surface and decrease lung inflammation	Luciani et al. (2012)
	Cftr^F508del/F508del^, cftr ^-/-^ and Cftr^F508/-^ mice, Cftr^F508/+^ and Cftr^F508del/F508del^ mice in *Becn1* haploinsufficient background (Becn1^+/−^)	Cystamine plus epigallocatechin gallate restore CFTR function and reduce lung inflammation in Cftr^F508del/F508del^ and Cftr^F508/-^ mice. ∆F508-CFTR rescue is linked to autophagy restoration, i.e., no rescue in Becn1^+/−^ background	Tosco et al. (2016)
	Cftr^F508del/F508del^ mice (129/FVB outbred background) and wt littermates	TG2 inhibition by cystamine restores PPARγ levels and nuclear localization, and reduces TNFα in lungs of CF mice	Luciani et al. (2009)
Deregulated nutrient sensing	Young adult CF mice homozygous for F508del-CFTR and wt litter mates	Reduced FOXO1 in muscle, but not in liver and adipose tissue of CF mice. Insulin-like growth factor 1 (IGF-1) increases FOXO1 in CF muscle tissue similar to the wt level, and increases it in adipose tissue of both mouse models	Smerieri et al. (2014)
Mitochondrial dysfunction	CF mouse models (Cftr^tm1kth^, Cftr^tm2Mrc^, and Cftr^tm1Unc^ in C57BL/6J background)	Reduced Nrf2-CFTR colocalization in CF mouse models with concomitantly reduced expression of Nrf2 target geneses HMOX1, NQO1, and GCLC.	Borcherding et al. (2019)
Cellular senescence	Nfkb1^-/-^ mice (C57B1/6 background) and fibroblast cultures from ear clipping, p55^△ns/△ns^ mice, and late-generation (F3-F4) terc^−/-^ mice bred from B6/Cg-TERC^tm1Rdp^/J	Chronic, low-grade inflammation induces telomere dysfunction, senescence, impaired tissue regeneration and premature aging. Anti-inflammatory or antioxidant treatment blocks accumulation of telomere-dysfunctional senescent cells in nfkb1^-/-^ tissues	Jurk et al. (2014)
Altered cellular communication	Nfkb1^-/-^ mice (C57B1/6 background) and fibroblast cultures from ear clippings, p55^△ns/△ns^ mice, and late-generation (F3-F4) terc^−/-^ mice bred from B6/Cg-TERC^tm1Rdp^/J	Chronic, progressive low-grade inflammation promoted by NF-κB can cause premature aging	Jurk et al. (2014)
	C57/B16 and NE^−/-^ mice	NE promotes CFTR degradation through activation of intracellular calpain protease causing loss of channel function	Le Gars et al. (2013)

Human studies			
Hallmark of aging	Study design	Main findings	Ref
Genomic instability	20 years follow up of CF patients receiving care in United States CF care center programs comparing their cancer incidence with that of the general population	Increased risk for lymphoid leukemia, testicular cancer and digestive tract (esophago-gastric junction, biliary tract, small bowel and colon) cancers in non-transplanted patients compared to the general population. Particularly high risk for digestive tract (mostly bowel) cancers in transplanted patients	Maisonneuve et al. (2013)
	*Cancer* incidence in CF and non-CF lung transplant recipients compared to the general population based on the United States transplant and 16 cancer registries	Overall cancer risk in CF lung transplant recipients is more increased than in non-CF recipients, particularly for colorectal cancer, esophageal cancer and non-Hodgkin lymphoma	Fink et al. (2017)
	CFTR expression in non-small cell lung cancer (NSCLC) and normal lung samples	Downregulated CFTR expression in NSCLC samples; correlation of low CFTR expression with advanced stage, lymph node metastasis and poor prognosis	Li et al. (2015)
	Urinary 8-hydroxydeoxyguano-sine (oh^8^dG, marker of free radical-induced DNA damage) from CF patients and age matched healthy controls and correlation with clinical status	Significantly higher urinary oh^8^dG in CF group, but no correlation with markers of lung function (FEV_1_, FVC) or clinical status assessed by Taussig-Schwachman score in CF patients; highly significant, positive correlation between urinary oh^8^dG and plasma vitamin E	Brown et al. (1995)
	Bronchoalveloar lavage (BAL) of CF and nonsmoking control subjects	Glutathione deficiency with significant reduction in GSH in CF BAL fluid. Simultaneously marked deficiency of plasma GSH.	Roum et al. (1985)
	BAL of children with CF and normal control subjects	Highest oxidative stress level assessed by protein carbonyls in patients with FEV1 < 80% of predicted or highly elevated neutrophils	Starosta et al. (2006)
Telomere attrition	Telomere length of DNA extracted from airway epithelial cells of CF patients and controls	No significant difference in telomere length between CF and control airway epithelial cells, apart for a small subgroup of CF subjects showing shorter telomeres	Fischer et al. (2013)
	Telomere length of DNA extracted from peripheral blood leukocyte of CF patients	Decreased telomere length associated with severe disease characterized by lower FEV_1_, ∆F508 homozygosity and CF asthma	Lammertyn et al. (2017)
	Telomere length of DNA extracted from tissue cores of healthy and diseased human lungs from (re-)transplantation or autopsy	Decreasing telomere length with age in normal, but not in CF lungs. No reduction in telomere length in CF explant lungs compared to normal lungs and no correlation between telomere length and disease severity	Everaerts et al. (2018)
Epigenetic alterations	DNA methylation profiling in nasal epithelial cells and whole blood from CF and control subjects	Substantial DNA methylation differences between CF and control samples, and different methylation levels between mild and severe CF disease. DNA methylation changes in genes responsible for epithelial integrity, inflammatory and immune response, and over-represented in enhancers active in lung tissue	Magalhães et al. (2018)
	DNA methylation profiling in BAL cells, i.e., primarily lung macrophages, from CF and normal subjects	Multiple differentially methylated CpG sites in CF BAL cells, mostly hypo-methylated and located in non-promoter CpG island as well as putative enhancer regions and DNase hypersensitive regions. Altered DNA methylation significantly associated with CF disease status and may be a driving factor in CF innate immune dysfunction	Chen et al. (2018)
Loss of proteostasis	Single-center, open-label phase-2 clinical trial with CF patients orally treated with cysteamine and epigallocatechin	Beneficial effects of proteostasis regulators in patients with rescuable CFTR with decreased sweat chloride, increased CFTR expression and function, restored autophagy, decreased sputum CXCL8 and TNF-α and tendency for improved respiratory function	Tosco et al. (2016)
Cellular senescence	Immunohistochemistry for senescence markers on tissue sections of lungs from CF patients and normal controls	Significantly increased expression of senescence/DNA damage markers (p16^INK4a^, γH2A.X and phospho-Chk2) in CF airways	Fischer et al. (2013)
Stem cell exhaustion	Bronchial epithelial cells from lung brush biopsies and blood neutrophils from CF patients homozygous for ∆F508 mutation and normal controls	miRNA-155, upregulated in aging and suspected to contribute to inflammation-associated stem cell dysfunction (Teramura and Onodera, 2018), is highly expressed in CF lung epithelial cells and blood neutrophils, where it reduces SHIP1 levels promoting PI3K/Akt activation that drives IL-8 expression	Bhattacharyya et al. (2011)
Altered cellular communication	TGF-β_1_ gene polymorphism in CF patients homozygous for ∆F508	Patients with high levels of the profibrotic, pro-aging cytokine TGF-β_1_ show accelerated lung function decline	Arkwright et al. (2000)
	TGF-β_1_ level in BAL fluid of pediatric CF patients and non-CF controls, and assessment of clinical disease indicators	Elevated TGF-β_1_ in CF patients is associated with neutrophilic inflammation, diminished lung function and recent hospitalization. No association of TGF-β_1_ with presence or quantity of bacterial pathogens	Harris et al. (2009)
	Estimation of lung volumes and forced expiratory flows in children before clinically indicated BAL	TGF-β_1_ and matrix metalloprotease (MMP)-2, both previously linked to airway remodeling, are associated with diminished flows as well as hyperinflation and air trapping	Peterson-Carmichael et al. (2009)
ECM dysregulation	Matrix metalloprotease (MMP) profile in lower airway secretions of CF in- and outpatients and normal controls	Increased active MMP-9 and NE, and decreased tissue inhibitor of metalloprotease-1 (TIMP-1) in CF. NE can activate pro-MMP-9 and degrade TIMP-1, hence not surprisingly a strong correlations between NE and MMP-9 activity in CF inpatient samples	Gaggar et al. (2007)
	BAL and endobronchial biopsies in children with CF and controls without lower airway symptoms	Increased elastin, glycosaminoglycan and collagen in BAL fluid from children with CF indicating matrix breakdown that positively correlates with age, neutrophil count and protease concentration. Increased reticular basement membrane thickness in CF group correlates with TGF-β_1_ level in BAL fluid	Hilliard et al. (2007)

## Conclusion and Further Directions

Increasing evidence suggests that accelerated aging processes are involved in CF lung pathology. Based on the current state of research summarized in the paragraphs above, it appears sufficiently established that accelerated aging in CF patients is not only a consequence of chronic lung inflammation, but aging-associated processes are also driving disease progression. Of the ten hallmarks of aging, according to recent literature all but stem cell depletion and telomere attrition appear to be involved to varying degrees in CF lung disease (cp. Figure 1). For some hallmarks there is already considerable data on the specific mechanisms of their pathologic involvement, as in the case of altered proteostasis and intercellular communication. Others, such as mitochondrial dysfunction, deregulated nutrient sensing and cellular senescence, have been indicated to play a role in CF lung pathology and accelerated aging, but further research is needed to elucidate underlying pathomechanisms. And although each of the ten hallmarks was discussed individually, they should be considered as interdependent processes that influence each other.

Growing evidence on the role of accelerated aging processes in CF lung disease and progress in deciphering involved mechanisms also provide new therapeutic targets for future treatment strategy complementing established therapies as summarized in *Therapeutic Implications*. Some therapies targeting the ten hallmarks of aging are already in clinical use, and further can be expected to follow which may aid further reducing mortality and improving quality of life in CF patients.

## Abbreviations

BAL, bronchoalveolar lavage; BK, large-conductance Ca2+- and voltage-dependent K+; ECM, extracellular matrix; FEV1, forced expiratory volume in 1s; FVC, forced ventilator capacity; GSH, reduced glutathione; HBE, human bronchial epithelial; IGF-1, Insulin-like growth factor 1; MAPK, mitogen-activated protein kinase; NE, neutrophil elastase; oh8dG, urinary 8-hydroxydeoxyguanosine; PBMCs, peripheral blood mononuclear cells; PMN, polymorphnuclear leukocyte; ROS, reactive oxygen species; TG2, tissue trans-glutaminase 2; TIMP-1 tissue inhibitor of metalloprotease-1; wt, wild-type.
